# Justice involvement patterns, overdose experiences, and naloxone knowledge among men and women in criminal justice diversion addiction treatment

**DOI:** 10.1186/s12954-019-0317-3

**Published:** 2019-07-16

**Authors:** Rachel E. Gicquelais, Briana Mezuk, Betsy Foxman, Laura Thomas, Amy S. B. Bohnert

**Affiliations:** 1 Department of Epidemiology, University of Michigan School of Public Health, 1415 Washington Heights, Ann Arbor, MI 48109 USA; 2 Department of Epidemiology, Johns Hopkins Bloomberg School of Public Health, 615 N. Wolfe Street, Baltimore, MD 21205 USA; 3 Department of Psychiatry, University of Michigan, 2800 Plymouth Road, Ann Arbor, MI 48109 USA; 4 Veterans Affairs Center for Clinical Management Research, 2215 Fuller Road, Ann Arbor, MI 48105 USA; 5 Current Address: Johns Hopkins Bloomberg School of Public Health, 615 N. Wolfe St, E7133A, Baltimore, MD 21205 USA

**Keywords:** Opioids, Overdose, Naloxone, Addiction treatment, Criminal justice system involvement, Latent class analysis

## Abstract

**Background:**

Persons in addiction treatment are likely to experience and/or witness drug overdoses following treatment and thus could benefit from overdose education and naloxone distribution (OEND) programs. Diverting individuals from the criminal justice system to addiction treatment represents one treatment engagement pathway, yet OEND needs among these individuals have not been fully described.

**Methods:**

We characterized justice involvement patterns among 514 people who use opioids (PWUO) participating in a criminal justice diversion addiction treatment program during 2014–2016 using a gender-stratified latent class analysis. We described prevalence and correlates of naloxone knowledge using quasi-Poisson regression models with robust standard errors.

**Results:**

Only 56% of participants correctly identified naloxone as an opioid overdose treatment despite that 68% had experienced an overdose and 79% had witnessed another person overdose. We identified two latent justice involvement classes: low involvement (20.3% of men, 46.5% of women), characterized by older age at first arrest, more past-year arrests, and less time incarcerated; and high involvement (79.7% of men, 53.5% of women), characterized by younger age at first arrest and more lifetime arrests and time incarcerated. Justice involvement was not associated with naloxone knowledge. Male participants who had personally overdosed more commonly identified naloxone as an overdose treatment after adjustment for age, race, education level, housing status, heroin use, and injection drug use (prevalence ratio [95% confidence interval]: men 1.5 [1.1–2.0]).

**Conclusions:**

All PWUO in criminal justice diversion programs could benefit from OEND given the high propensity to experience and witness overdoses and low naloxone knowledge across justice involvement backgrounds and genders.

**Electronic supplementary material:**

The online version of this article (10.1186/s12954-019-0317-3) contains supplementary material, which is available to authorized users.

## Background

Mortality from opioid overdose quadrupled from 1999 to 2016 in the United States (US) [1, 2]. To reduce overdose mortality, there is a critical need for overdose education and naloxone distribution (OEND) programs to identify, engage, and train people who use opioids (PWUO), as they are both potential overdose victims and bystanders who could respond [3–5]. OEND programs train potential overdose bystanders to identify and respond to an opioid overdose and equip participants with naloxone, an opioid antagonist that reverses the respiratory depression caused by high doses of opioids [6, 7]. Many also provide information about Good Samaritan Laws, which protect persons present at an overdose from legal prosecution for illegal activities discovered when they call 911 [7]. Naloxone distribution to PWUO is cost-effective, especially when combined with addiction treatment, and reduces opioid overdose mortality [8–10]. However, as highlighted recently by the US Surgeon General [11], there remains an urgent need to maximize the number of PWUO who are well-positioned to respond to an overdose or benefit from receipt of naloxone.

OEND programs are increasingly incorporated into addiction treatment services, jails, and prisons given the high prevalence of PWUO in these settings and the elevation in overdose risk following addiction treatment and incarceration [4, 12–14]. Mortality among PWUO is up to 21-fold higher after addiction treatment and up to 129-fold higher in the weeks after incarceration relative to the general population [12, 15]. The elevated risk of overdose during these periods is due to a loss of physiologic tolerance to opioids during periods of incarceration or treatment [16]. A lack of access to medication-assisted treatments and social services (e.g., housing) may further contribute to a return to opioid use and subsequent heightened overdose risk [12]. Pre-release naloxone distribution in corrections institutions has reduced population-level overdose risk [17] and may have benefits for witnessed overdose [18].

The US Surgeon General recently called for an improvement in the pre- and post-release addiction treatment services available to incarcerated PWUO and for a “transition to a less punitive and more health-focused approach” [11]. Herein, we examine one less punitive approach in which the addiction treatment and criminal justice system settings intersect: justice diversion addiction treatment programs, which provide PWUO facing legal prosecution with addiction treatment to reduce sentences or avoid criminal charges [13, 19, 20]. PWUO are referred to justice diversion programs by law enforcement, drug courts, the correctional system, or through parole or probationary boards [19, 21–25]. How best to tailor OEND programs to minimize post-treatment overdose risk among clients of justice diversion addiction treatments has not been thoroughly examined.

This study aims to inform OEND planning using a sample of 514 PWUO in a residential justice diversion addiction treatment program in Michigan. First, we characterize justice involvement preceding diversion (e.g., arrest history, age at first arrest, time incarcerated), history of overdose experiences and witnessed overdose, and naloxone knowledge among PWUO in a justice diversion addiction treatment program. We evaluate these separately by gender to account for potential differences in justice involvement for men and women. Second, we evaluate whether justice involvement history is associated with experiencing or witnessing an overdose, given that prior research has suggested a relationship between criminal justice involvement and overdose risk [3, 5, 23, 26–28]. Finally, we examine the relationship of overdose experiences and justice involvement with naloxone knowledge. Based on prior literature, we hypothesized that we would identify subgroups with higher intensities of involvement that could benefit from targeted OEND due to their low naloxone awareness [23, 27–31]. We also hypothesized that, consistent with prior research, personally experiencing and witnessing an overdose would be associated with higher naloxone knowledge [32–34].

## Methods

### Study description

The analytic sample was drawn from a previously described study of 817 adult (≥ 18 years) patients receiving treatment for drug or alcohol use disorders in a residential addiction treatment program located in a suburban area of Southeast Michigan during October 2014–January 2016 [35]. This facility served patients living throughout Michigan and received client referrals through contracts with the Michigan Department of Corrections. The typical treatment duration for patients was 60–90 days and patients were separated by gender. Research assistants approached eligible patients (who were aged ≥ 18 years and able to provide informed consent) about their interest in completing a self-administered survey to assess eligibility for enrollment in a randomized controlled trial. Interested participants provided informed consent, completed a paper and pencil survey that took approximately 1 h to finish, and received $5 for participating. This analysis uses data from the cross-sectional survey and is not restricted to those who participated in the randomized controlled trial. The University of Michigan Institutional Review Board approved the study protocol.

We restricted the analytic sample to participants whose treatment was prompted by the justice system (excluded *n* = 40 participants), had used opioids (heroin or prescription opioids not prescribed by a doctor) in their lifetime (excluded *n* = 237 participants), and who had non-missing responses to the measures described below (excluded *n* = 26 participants, see Additional file 1: Figure S1). Our analytic sample included 514 PWUO.

### Measures

#### Justice involvement

We quantified participants’ cumulative and recent pre-treatment justice system interactions using five items: age at first arrest (median 18, range 9–59 years), number of past-year arrests (median 1, range 0–42 arrests), number of lifetime arrests (mode 6–10, categories 1–2, 3–5, 6–10, 11–49, 50–99, or 100 or more arrests), number of months during the past year spent in jail or prison (median 5.3, range 0–12 months), and lifetime number of years spent in jail or prison (median 3.5, range 0–41.3 years). We formed categorical variables using quartile or tertile breaks from distributions in the analytic sample, with modifications when appropriate to enhance interpretability (e.g., juvenile versus adult age at first arrest). Categorical variables included age at first arrest (9–17, 18–20, or 21–59 years), past-year arrests (0, 1–2, 3–42), lifetime arrests (1–5, 6–10, ≥ 11), past-year time spent in jail or prison (0–1.9, 2–5.9, 6–10.9, 11–12 months), and total time spent in jail or prison (0–0.9, 1–3.4, 3.5–7.4, 7.5–41.3 years).

#### Personal overdose experiences and witnessed overdose

Before answering questions, participants read the following definition of an overdose: “The following questions are about experiences with taking too much drugs or medications/pills. This is sometimes called ‘poisoning,’ ‘nodding out,’ or an ‘overdose’ or ‘OD.’” Participants reported the number of overdoses experienced, timing of their most recent overdose, and substances used during the most recent overdose. Participants then read the definition of a witnessed overdose: “The following questions are about times you have seen someone else taking too much drugs or medications/pills, and/or drinking too much alcohol. This is sometimes called an ‘overdose.’ When someone has an overdose, they might have blue skin color, convulsions, or difficulty breathing, lose consciousness, collapse, cannot be woken up, or have a heart attack or die.” [36] and reported the number of overdoses they witnessed and drugs used by the victim during the most recently witnessed overdose. We formed binary variables for ever experiencing an overdose, experiencing an overdose in the past year, and ever witnessing an overdose. We assessed the number of lifetime personal and witnessed overdoses as three-level categorical variables (0, 1–5, or ≥ 6) and summarized whether the participant’s most recent overdose experience and witnessed overdose involved heroin or prescription opioids.

#### Covariates

Participants reported whether they had heard of naloxone and identified its purpose as an overdose treatment, drug treatment for opioid dependence, detox, other, or do not know (multiple responses were allowed). For the analysis, we defined naloxone knowledge as having heard of naloxone *and* correctly identifying its purpose as an overdose treatment. We also examined demographic characteristics, including age (18–29, 30–44, 45–67 years), housing (dichotomized into temporary housing [rooming house/hotel, halfway house/group home, inpatient treatment facility/hospital, jail, shelter, or homeless] vs. stable housing [house/apartment or friend/family member’s house]), education (less than high school/GED or high school/GED or higher), race (black, white, other, or multiple), and ethnicity (Hispanic vs. non-Hispanic). We also summarized substance use characteristics in several time frames, including lifetime and past-year heroin and illicit prescription opioid use (defined as use that was not as prescribed by a doctor). Additionally, we summarized whether participants used heroin for ≥ 7 consecutive days or injected any substance in the month prior to entering treatment or jail. Finally, we described nonmedical prescription opioid use in the month before entering treatment or jail using four items from the Current Opioid Misuse Measure found to describe nonmedical prescription opioid use in the addiction treatment setting [37, 38]. Specifically, we summarized whether participants reported engaging in any of the following when using prescription opioids: taking prescription opioids belonging to someone else, borrowing prescription opioids from someone else, using more than they were prescribed, or using prescription opioids to treat symptoms other than pain.

### Latent class analysis

#### Latent class measurement model

Latent class analysis (LCA) is a statistical technique used to describe unobserved (i.e., latent) subgroups from patterns of observed variables [39]. It is helpful for identifying clusters (subgroups) of individuals who share patterns of characteristics. Lorvick et al. previously described three classes of justice involvement (low, medium, and high) among women who used drugs in California based on their incarceration history and community corrections involvement [29]. We used LCA to identify subgroups of criminal justice system involvement based on five categorical variables: age at first arrest, past year arrests, lifetime arrests, past year time spent in jail or prison, and total time spent in jail or prison.

We fit LCA models with two to six classes and selected the number of latent classes using a combination of interpretability and model fit indices (Akaike information criterion [AIC], Bayesian information criterion [BIC], adjusted BIC, and entropy). Smaller values of the AIC and BIC, and larger values of entropy indicate better relative model fit [39]. After selecting the number of classes, we ensured convergence to a globally optimal solution using 1000 random start values. Item response probabilities, which reflect the distribution of each observed justice involvement variable within each justice involvement class, provided the basis for investigator-assigned class labels used to describe each latent class. We completed LCA analyses in SAS version 9.4 using PROC LCA [39].

#### Justice involvement by gender

Men and women have different criminal sentencing patterns [40], and the relationship of offenses with drug-related mortality differs by gender [31]. Additionally, men and women are treated separately in many residential addiction treatment programs, including the facility where these data were collected. Therefore, we assessed whether the justice involvement measurement model operated similarly in groups defined by gender (men vs. women). We fit the LCA model with and without constraints that required item response probabilities to be equal by gender, testing the null hypothesis of measurement invariance (i.e., that item response patterns were the same for men and women) [39]. We used a likelihood ratio test (LRT) to test for measurement invariance. Rejecting the LRT (*p* < 0.05) implied that the measurement model differed by gender.

### Correlates of overdose experience, witnessed overdose, and naloxone knowledge

We examined whether the prevalence of experiencing or witnessing an overdose differed by justice involvement class. We also assessed whether naloxone knowledge was associated with ever experiencing or witnessing an overdose or with justice involvement. We summarized associations using bivariate and adjusted prevalence ratios from quasi-Poisson regression models with robust standard errors, an approach appropriate for highly prevalent binary outcomes [41, 42]. Adjusted models included sociodemographic characteristics (age, race, housing status, education level) and substance use characteristics (heroin use and injection drug use), as these covariates could be associated with naloxone knowledge or related outcomes and the main exposures for this analysis (overdose, witnessed overdose, and justice involvement) [5, 32, 33, 43, 44]. For regression analyses, we formed a categorical justice involvement variable by assigning participants to their most likely latent justice involvement class (i.e., the modal class assignment approach).

### Sensitivity analyses

We conducted two sensitivity analyses. First, to assess whether the relationships between justice involvement and experiencing an overdose, witnessing an overdose, and naloxone knowledge were robust to the modal class assignment LCA approach, we used the pseudo-class draws approach [45]. We conducted 20 imputations that each assigned participants to a justice involvement class based on LCA posterior probabilities [45]. We repeated quasi-Poisson regressions for each imputed dataset for all associations between justice involvement and overdose outcomes that reached statistical significance using the modal class assignment approach and pooled results using imputation procedures [46]. Second, to examine whether our findings were similar among people who had used opioids recently relative to when they entered treatment, jail, or prison, we re-analyzed the relationships between justice involvement, experiencing an overdose, and witnessing an overdose with naloxone knowledge after restricting the sample to participants who reported using heroin or prescription opioids not prescribed to them in the past year and/or who reported using prescription opioids nonmedically in the month before entering treatment or jail.

## Results

### Participant characteristics

Most participants were white (74.7%), non-Hispanic (95.3%), and aged 30–44 years (Table 1). Nearly half were arrested for the first time as juveniles (47.9%). Most were arrested once or twice in the year before treatment or jail (41.6%) while 32.5% had no arrests. Participants spent a median of 3.5 years in their lifetime and 5.3 months of the past year incarcerated.

**Table 1:** Sample description of 514 people who use opioids in justice diversion addiction treatment during 2014–2016 by gender

	Total	Women	Men
	*n* (%)	*n* (%)	*n* (%)
Total	514 (100)	151 (100)	363 (100)
Justice involvement^a^			
Age at 1st arrest (years)			
Missing	2 (0.4)	0 (0)	2 (0.6)
9–17	246 (47.9)	46 (30.5)	200 (55.1)
18–20	138 (26.9)	48 (31.8)	90 (24.8)
21–59	128 (24.9)	57 (37.8)	71 (19.6)
Median (IQR)	18 (16–20.5)	19 (17–22)	17 (15–19)
Lifetime arrests			
Missing	0 (0)	0 (0)	0 (0)
1–5	173 (33.7)	61 (40.4)	112 (30.9)
6–10	171 (33.3)	49 (32.5)	122 (33.6)
11 or more	170 (32.1)	41 (27.2)	129 (35.5)
Arrests in year before treatment or jail			
Missing	3 (0.6)	0 (0)	0 (0)
0	167 (32.5)	24 (15.9)	143 (39.4)
1–2	214 (41.6)	77 (51.0)	137 (37.7)
3–42	130 (25.3)	50 (33.1)	80 (22.0)
Median (IQR)	1 (0–3)	2 (1–3)	1 (0–2)
Time spent in jail or prison in lifetime (years)			
Missing	7 (1.4)	4 (2.7)	3 (0.8)
0–0.9	107 (20.8)	66 (43.7)	41 (11.3)
1–3.4	135 (26.3)	47 (31.1)	88 (24.2)
3.5–7.4	134 (26.1)	23 (15.2)	111 (30.6)
7.5–41.3	131 (25.5)	11 (7.3)	120 (33.1)
Median (IQR)	3.5 (1–7.5)	1.1 (0.3–3)	5 (2.3–9.8)
Time spent in jail or prison in past year (months)			
Missing	17 (3.3)	8 (5.3)	9 (2.5)
0–1.9	119 (23.2)	43 (28.5)	76 (20.9)
2–5.9	134 (26.1)	59 (39.1)	75 (20.7)
6–10.9	125 (24.3)	28 (18.5)	97 (26.7)
11–12	119 (23.2)	13 (8.6)	106 (29.2)
Median (IQR)	5.3 (2–10)	3.1 (1.4–6)	6.5 (2.9–12)
Overdose experience			
Experienced an overdose	350 (68.1)	114 (75.5)	236 (65.0)
Most recent overdose involved heroin and/or prescription opioids^b^	254 (72.6)	87 (76.3)	167 (70.8)
Experienced an overdose in the year before treatment	219 (42.7)	83 (55.0)	136 (37.5)
Number of experienced overdoses in lifetime			
0	164 (31.9)	37 (24.5)	127 (35.0)
1–5	225 (43.8)	63 (41.7)	162 (44.6)
6 or more	125 (24.3)	51 (33.8)	74 (20.4)
Witnessed overdose			
Witnessed any overdose	407 (79.2)	127 (84.1)	280 (77.1)
Most recently witnessed overdose involved heroin and/or prescription opioids^c^	339 (83.3)	117 (92.1)	222 (79.3)
Number of witnessed overdoses in lifetime			
0	107 (20.8)	24 (15.9)	83 (22.9)
1–5	269 (52.3)	84 (55.6)	185 (51.0)
6 or more	138 (26.9)	43 (28.5)	95 (26.2)
Naloxone knowledge			
Heard of naloxone	319 (62.1)	109 (66.9)	210 (57.9)
Identified purpose of naloxone^d^	289 (90.6)	101 (92.7)	188 (89.5)
Demographic and social characteristics			
Age (years), Median (IQR)	34 (27–46)	31 (26–40)	36 (28–48)
Race			
Black	83 (16.2)	18 (11.9)	65 (17.9)
White	384 (74.7)	116 (76.8)	268 (73.8)
Other	13 (2.5)	3 (2.0)	10 (2.8)
Multiple races	34 (6.6)	14 (9.3)	20 (5.5)
Hispanic ethnicity	24 (4.7)	5 (3.3)	19 (5.2)
Less than high school education/GED	83 (16.2)	54 (14.9)	29 (19.2)
Temporary housing in past 3 months^e^	290 (56.4)	77 (51.0)	213 (58.7)
Substance use			
Lifetime heroin use	347 (67.5)	117 (77.5)	230 (63.3)
Heroin use in the past year^f^	249 (71.9)	93 (79.5)	156 (67.8)
Used heroin ≥ 7 consecutive days during the month before treatment or jail^f^	194 (55.9)	70 (59.8)	124 (53.9)
Lifetime prescription opioid use (not as prescribed a doctor)	485 (94.4)	144 (95.4)	341 (93.9)
Used prescription opioids in the past year (not as prescribed by a doctor)^g^	271 (55.9)	96 (66.7)	175 (51.3)
Took or borrowed prescription opioids belonging to someone else, took more than prescribed, or used for reasons other than for pain management	371 (72.2)	118 (78.1)	253 (70.0)
Injected drugs in the month before entering treatment or jail	221 (43.0)	75 (49.7)	146 (40.2)

^a^Latent class analysis allows for missing values in indicators and uses information on available indicators to create classes for participants with missing data. Therefore, totals for justice involvement may not add to the full sample size

^b^Among those who experienced an overdose. Includes most recent experienced overdose events where the participant reported they used heroin and/or prescription opioids. An additional 7 participants (5 men, 2 women) did not report substances used

^c^Among those who witnessed an overdose. Includes most recently witnessed overdose events where the participant reported that the victim used heroin and/or prescription opioids. An additional 9 participants (6 men, 3 women) did not know or did not report substances used by the victim

^d^Among those who had heard of naloxone

^e^Includes living in a halfway house or group home, inpatient facility, jail, shelter, or homeless

^f^Among those who used heroin in their lifetime. An additional 16 participants (10 men, 6 women) reported lifetime heroin use but declined to answer questions about past year heroin use. An additional 5 participants (4 men, 1 woman) declined to answer questions about use in the 30 days before entering treatment or jail

^g^Among those who used prescription opioids in their lifetime. An additional 19 participants (14 men, 5 women) reported lifetime prescription opioid use but declined to answer questions about past year prescription opioid use

Most participants had experienced (68.1%) and/or witnessed (79.2%) an overdose, and 42.7% overdosed in the past year. Only 56.2% of participants had naloxone knowledge (62.1% had heard of naloxone and 90.6% of those who had heard of it correctly identified it as an overdose treatment).

### Gender-stratified justice involvement LCA measurement model

While the BIC indicated optimal fit for a three-class justice involvement LCA model, the two-class model had higher entropy, larger and more stable classes, and was more interpretable than other models (Table 2). Descriptive analysis suggested that justice involvement characteristics differed by gender (Table 1), and we rejected the null hypothesis of measurement invariance using the LRT and the two class model (χ^2^ = 72.0, degrees of freedom: 24, *p* value < 0.05), implying that item response probabilities and latent class interpretations differed by gender. Therefore, we used the two-class gender-stratified model for the remainder of analyses.

**Table 2:** Fit of latent classes models of justice involvement among a sample of people who use opioids in justice diversion addiction treatment during 2014–2016 (*n* = 514)

Classes	Log likelihood	AIC	BIC	Adjusted BIC	Entropy
2	− 2897.1	595.7	701.7	622.4	*0*.*82*
3	− 2852.7	532.9	*694*.*1*	*573*.*5*	0.69
4	− 2841.6	536.7	753.0	591.1	0.71
5	− 2822.0	*523*.*5*	795.0	591.8	0.73
6	**−** *2809*.*3*	524.1	850.8	606.4	0.73

Italic font indicates optimal fit index value of the tested solutions

**Fig. 1:**
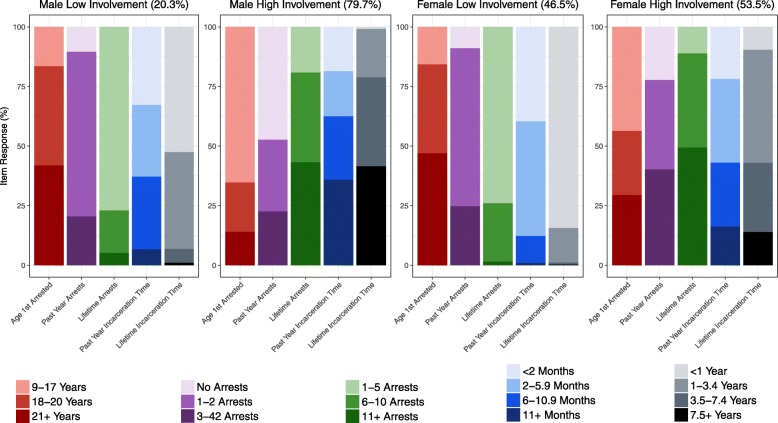
Patterns of justice involvement among men and women who use opioids in justice diversion addiction treatment during 2014–2016 (*n* = 363 men and 151 women). Two justice involvement classes per gender were identified among a sample of 514 PWUO in justice diversion addiction treatment. Men with low involvement (20.3% of men) were arrested for the first time at an older age and arrested more often in the past year. Men with high involvement (79.7%) had more arrests and incarceration time. Similar classes emerged among women, but women had more past year arrests and spent less time incarcerated than men. Women with low involvement comprised 46.5% of the sample and high involvement was slightly more common (53.5%)

The gender-stratified model recovered two justice involvement classes for each gender that we termed “high” and “low” involvement (Fig. 1). Men with low justice involvement (20.3% of men) had an older age at first arrest (median 19, mean 22.2 years), few lifetime arrests (80.6% had 1–5 arrests), and less incarceration time (lifetime median 0.8, mean 1.2 years; past year median 4.0, mean 4.3 months); 72.2% had 1–2 arrests in the year before treatment. Men with high justice involvement (79.7% of men) were more commonly arrested for the first time as a juvenile (65.3%), had more past year (median 8, mean 7.2 months), and lifetime incarceration time (median 6, mean 8.3 years), and had more lifetime arrests (81.4% had six or more lifetime arrests).

The defining features and item response probabilities among women differed from men. Women with low justice involvement (46.5% of women) were more likely to be arrested at an older age at first arrest (84.2% aged ≥ 18 years), had few lifetime arrests (75.7% with 1–5 arrests), and spent less time incarcerated (lifetime median 0.3, mean 0.5 years; past year median 2.6, mean 2.8 months). Women with high justice involvement (53.5% of women) were younger at their first arrest (70.3% < 21 years), had more lifetime arrests (50.6% had ≥ 11 arrests), and spent more time incarcerated (lifetime median 4.4, mean 2.9 years; past year median 4, mean 5.4 months).

### Correlates of overdose and naloxone knowledge

**Fig. 2:**
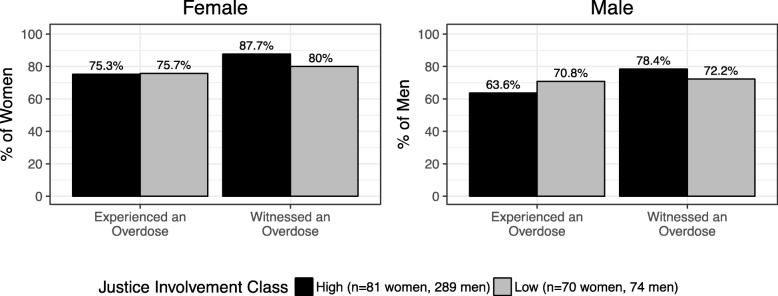
Prevalence of experiencing and witnessing an overdose among people who use opioids in justice diversion addiction treatment during 2014–2016 (*n* = 363 men and 151 women). Prevalence of experiencing and witnessing an overdose was high across justice involvement groups in both genders. Prevalence of overdose outcomes did not differ by justice involvement history

**Fig. 3:**
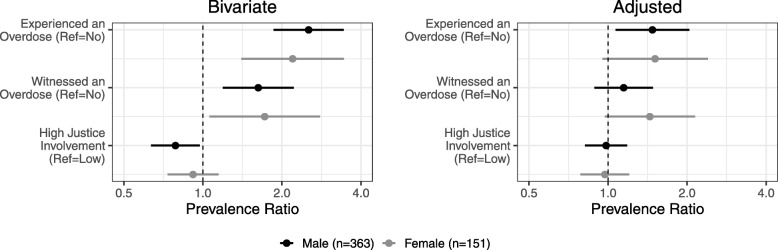
Associations of overdose experience, witnessing an overdose, and justice involvement with naloxone knowledge among men and women who use opioids in justice diversion addiction treatment during 2014–2016. Prevalence of naloxone knowledge was higher men who had experienced an overdose in their lifetime (adjusted prevalence ratio [aPR, 95% confidence interval, CI] men: 1.5 [1.1–2.0]) and marginally higher among women who had experienced an overdose (aPR [95% CI] 1.5 [0.95–2.4]). Women who had witnessed an overdose were also marginally more likely to have naloxone knowledge (aPR [95% CI] 1.4 [0.97–2.1]). There was no difference in prevalence of naloxone knowledge for men or women by their justice involvement history (aPR [95% CI] men 0.98 [0.82–1.2, women 0.97 [0.79–1.2]). Among men, there was also no difference in naloxone knowledge by history of witnessing an overdose (aPR [95% CI] 1.1 [0.89–1.5]). Adjusted prevalence ratios are adjusted for age, race, education level, residence in temporary housing (defined as reporting living in a halfway house or group home, inpatient facility, jail, shelter, or homeless), lifetime heroin use, and injection drug use in the 30 days prior to treatment. *Ref* reference group

We found no differences in prevalence of experiencing or witnessing an overdose by justice involvement in bivariate analyses (Fig. 2). Experiencing an overdose and witnessing an overdose were both positively associated with naloxone knowledge among men and women (Fig. 3). Only 26.0% of men who had never experienced an overdose were knowledgeable of naloxone, whereas 65.7% of men with lifetime overdose experience had naloxone knowledge. Among women, 35.1% of women who had not overdosed had naloxone knowledge vs. 77.3% who overdosed had naloxone knowledge. High justice involvement was associated with lower naloxone knowledge among men in bivariate analyses.

Men who experienced an overdose in their lifetime were 50% more likely to have naloxone knowledge than men who had not experienced an overdose after adjustment for age, race, education level, residence in temporary housing, lifetime heroin use, and injection drug use in the 30 days before treatment (PR [95% CI] 1.5 [1.1–2.0]). Women who experienced an overdose (PR [95% CI] 1.5 [0.95–2.4], *p* = 0.08) or witnessed an overdose (PR [95% CI] 1.4 [0.97–2.1], *p* = 0.07) in their lifetime were marginally more likely to have naloxone knowledge. Naloxone knowledge among men did not differ by justice involvement after adjustment (PR [95% CI] 0.98 [0.79–1.2]).

### Sensitivity analyses

Assigning justice involvement classes with multiple imputation (i.e., the pseudo-class approach) yielded similar results to modal class assignment. The bivariate association of justice involvement with naloxone knowledge among men was not statistically significant after multiple imputation (PR [95% CI] 0.79 [0.61–1.0], *p* = 0.08).

After restricting the analytic sample to participants who reported using heroin or prescription opioids not prescribed to them in the past year and/or who reported using prescription opioids nonmedically in the month before entering treatment or jail, 423 participants (288 men, 135 women) remained for analysis. Men who experienced an overdose remained more likely to have naloxone knowledge in adjusted analysis (PR [95% CI] 1.5 [1.1–2.2], Additional file 1: Table S1). The marginal relationships of experiencing and witnessing an overdose among women were no longer present.

## Discussion

The primary finding of this study was that nearly all PWUO receiving treatment at this residential justice diversion addiction treatment facility during 2014–2016 had experienced and/or witnessed an overdose, but only half had heard of naloxone and correctly identified it as an overdose treatment. Thus, PWUO receiving treatment at this facility are appropriate candidates for OEND given their high likelihood of witnessing or experiencing an overdose after treatment completion [3, 5]. Justice involvement was not associated with naloxone knowledge or with overdose experiences, either personally or as a witness. These findings suggest that clients in justice diversion residential treatment programs in Michigan may be candidates for diversion-based OEND, regardless of their path to treatment. Future work could examine whether OEND scale-up in diversion-based treatment facilities elsewhere should be similarly generalized to all clients or focus on particular PWUO or other subgroups receiving treatment.

In light of continued increases in overdose mortality, the US Surgeon General recently highlighted a need for comprehensive addiction treatment services in jails and prisons and a focus on criminal justice reforms that improve the health of PWUO [1, 2, 11]. We identified two subgroups of PWUO that were defined by simultaneously examining several aspects of their justice involvement history using LCA. These subgroups reflected two pathways that men and women were diverted to treatment in Michigan. Most men (80%) had long-term justice involvement, whereas few were diverted after many recent arrests, likely to avoid incarceration. Women had had more past year arrests and spent less time incarcerated than men. High and low justice involvement was equally common among women. The patterns of justice involvement in this study reflect both justice involvement patterns among all PWUO and the selection process for diversion programs, such as the one where this study was conducted. For criminal justice reforms to decrease post-incarceration overdose mortality, there is both a need to incorporate OEND into the justice diversion addiction treatment setting and to assess whether current policies divert PWUO at highest risk of returning to opioid use and overdosing after treatment. Whether current diversion program eligibility criteria exclude PWUO at highest risk of post-incarceration overdose given, for example, many recent arrests, is unknown.

The prevalence of experiencing and witnessing an overdose in our study approached the maximum estimates reported in a 2015 systematic review (i.e., 50–96% of people who use illicit drugs witness an overdose and 17–68% personally experience an overdose) [5]. The fact that just over half of participants had heard of naloxone and identified it as an overdose treatment, demonstrating lower awareness than has been documented in prior studies [32, 43, 44, 47], highlights the need for the educational component of OEND in this setting. Naloxone knowledge was particularly low among male participants who had never personally experienced an overdose. Results from prior research describing the relationship between personally experiencing an overdose and naloxone knowledge and carrying naloxone have been mixed [32, 48], though one study characterized an association between personal concern for overdose risk and accepting a supply of naloxone in the emergency department [33] and qualitative evidence suggests that PWUO may learn about naloxone from emergency medical providers who respond when they experience an overdose [34]. While we cannot comment on whether these individuals experienced an overdose post-treatment, the fact that they were in addiction treatment, had used opioids, and had no knowledge of naloxone implies that they would benefit from OEND during incarceration or treatment. We found no differences in prevalence of experiencing or witnessing an overdose or in naloxone knowledge by justice involvement, supporting that OEND should be provided to all PWUO in justice diversion addiction treatment.

Our study has several strengths. The LCA approach allowed for a synthesis of several aspects of justice involvement simultaneously. The two groups that emerged were not evident when we examined each justice involvement indicator in isolation. We also had a large sample of PWUO diverted to addiction treatment at several stages post-arrest, which encompassed a variety of potential clients eligible for justice diversion addiction treatment. Another benefit of the large sample size was our ability to stratify our analysis by gender.

Our findings are not without limitation. We studied participants from a single addiction treatment facility located in a suburban area of the mid-Western US and all received treatment because of their criminal justice system involvement. The prevalence of naloxone knowledge, experiencing an overdose, and witnessing an overdose may reflect levels of OEND implementation specific to the Midwest and may not be generalizable outside this region, given that the availability of OEND and other harm reduction services is known to vary geographically [49, 50]. We were unable to determine when clients were diverted relative to the time they committed the crimes preempting treatment and cannot comment on specific differences between those diverted after arrest, incarceration, or parole/probation. Given the variability in diversion programs, it is difficult to determine whether the patterns of justice involvement observed here would extend to other states [19]. Our ability to evaluate whether our results reflect trends in justice involvement in non-diverted PWUO is limited by the lack of published criteria for diversion program eligibility. This lack of objective criteria further limited our ability to disentangle the sources of gender and other disparities (e.g., by race) in diversion.

Our study relied on self-reported characteristics from the pre-treatment period, potentially introducing recall biases. The cross-sectional design limited our ability to define the temporal sequence of events (e.g., whether individuals experienced or witnessed overdoses after their involvement with the criminal justice system). We had no information about participants’ access to or experience with syringe services programs or other harm reduction programs or how long they had used opioids, both of which may impact their familiarity with naloxone. Our results may not be generalizable to persons who misused their own prescription opioids as we restricted the analytic sample to participants who self-reported ever using opioids (heroin or prescription opioids not prescribed by a doctor), which may have low sensitivity to misuse of one’s own prescribed opioids [51, 52]. Finally, although we had an appropriately high entropy (> 0.8) to assign individuals to their most probable justice involvement class, this approach may have underestimated the magnitude of associations between justice involvement with overdose, witnessed overdose, and naloxone knowledge [53–55].

## Conclusions

The low prevalence of naloxone knowledge and high prevalence of experiencing and witnessing an overdose in our sample of PWUO suggests that OEND should be routinely incorporated into justice diversion addiction treatment. Further, OEND should be provided to all clients, regardless of pre-treatment overdose experience or justice involvement characteristics.

## Additional file

Additional file 1: **Figure S1**. Inclusion Criteria: Selection of Participants for a Study of People who Use Opioids in Justice Diversion Addiction Treatment during 2014-2016. **Table S1**. Bivariate and Adjusted Logistic Regression Results for Correlates of Naloxone Knowledge among 423 Men and Women in Justice Diversion Addiction Treatment during 2014-2016 who Used Heroin or Prescription Opioids in the Past Year or who Misused Prescription Opioids in the 30 Days Prior to Jail, Prison, or Attending Treatment. (PDF 188 kb)

## Abbreviations

AIC: Akaike information criterion
BIC: Bayesian information criterion
CI: Confidence interval
LCA: Latent class analysis
LRT: Likelihood ratio test
OEND: Overdose education and naloxone distribution
PR: Prevalence ratio
PWUO: People who use opioids
US: United States
