# Supplementary material for: Justice involvement patterns, overdose experiences, and naloxone knowledge among men and women in criminal justice diversion addiction treatment
Source: Harm Reduct J. 2019 Jul 16;16:46. doi: 10.1186/s12954-019-0317-3 (PMC6636104; doi:10.1186/s12954-019-0317-3)
Supplement: Supplementary file 1 — Figure S1. Inclusion Criteria: Selection of Participants for a Study of People who Use Opioids in Justice Diversion Addiction Treatment during 2014-2016. Table S1. Bivariate and Adjusted Logistic Regression Results for Correlates of Naloxone Knowledge among 423 Men and Women in Justice Diversion Addiction Treatment during 2014-2016 who Used Heroin or Prescription Opioids in the Past Year or who Misused Prescription Opioids in the 30 Days Prior to Jail, Prison, or Attending Treatment. (PDF 188 kb) [file 12954_2019_317_MOESM1_ESM.pdf]

Additional File 1: Justice Involvement Patterns, Overdose Experiences, and Naloxone Knowledge among Men and Women in Criminal Justice Diversion Addiction Treatment

Rachel E. Gicquelais[a,b,c]*, Briana Mezuk[a], Betsy Foxman[a], Laura Thomas[c,d], Amy S.B. Bohnert[c,d]

[a]Department of Epidemiology, University of Michigan School of Public Health, 1415 Washington Heights, Ann Arbor, MI, 48109, USA

[b]Department of Epidemiology, Johns Hopkins Bloomberg School of Public Health, 615 N. Wolfe Street, Baltimore, MD, 21205, USA

[c]Department of Psychiatry, University of Michigan, 2800 Plymouth Road, Ann Arbor, MI, 48109, USA

[d]Veterans Affairs Center for Clinical Management Research, 2215 Fuller Road, Ann Arbor, MI 48105

*Corresponding author: rgicque1@jhu.edu

Additional File 1

Figure S1. Inclusion Criteria: Selection of Participants for a Study of People who Use Opioids in Justice Diversion Addiction Treatment during 2014-2016

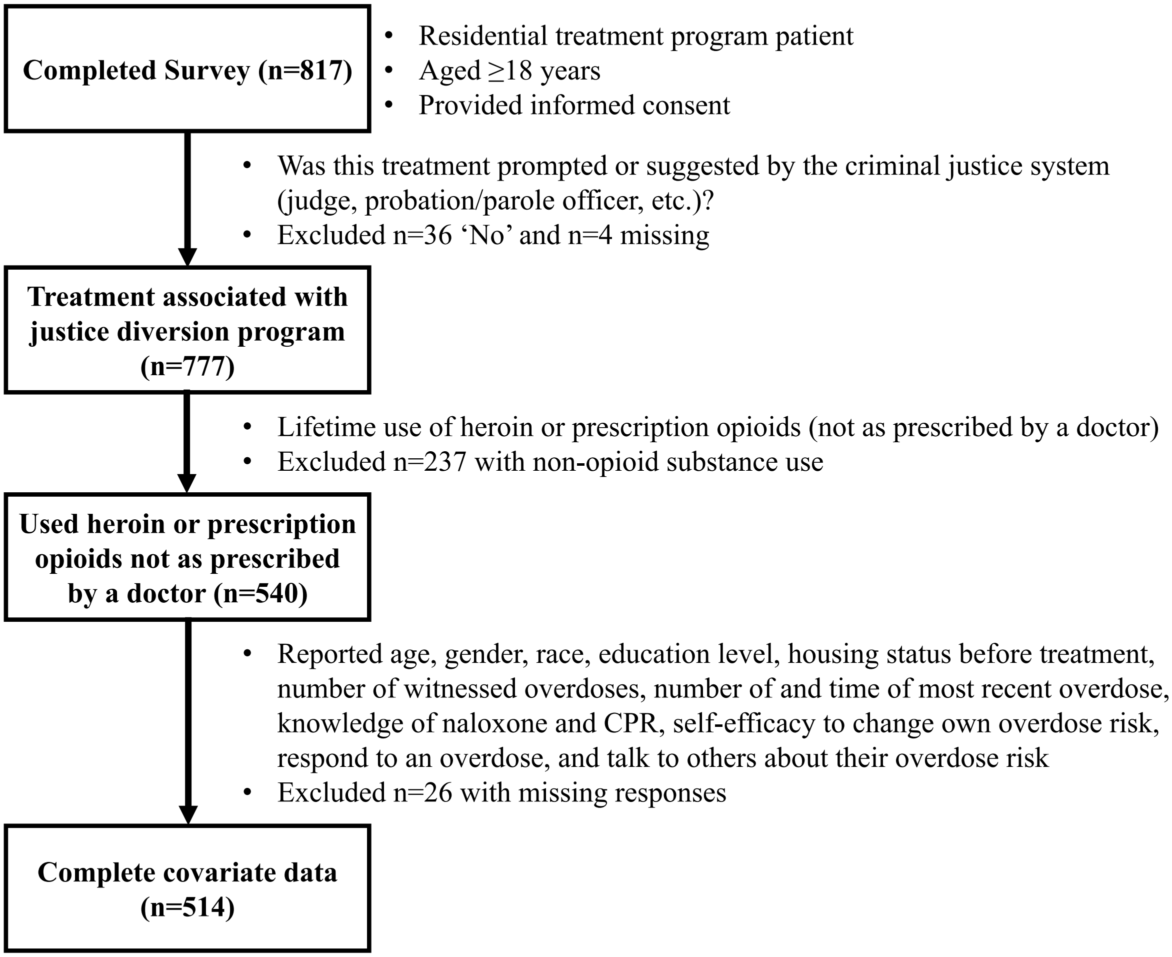

Our study included a sample of 514 PWUO in residential treatment who enrolled and consented to participate, affirmed their involvement in justice diversion, used heroin or prescription opioids (not as prescribed by a doctor) in their lifetime, and had complete data on covariates of interest.

Table S1. Bivariate and Adjusted Logistic Regression Results for Correlates of Naloxone Knowledge among 423 Men and Women in Justice Diversion Addiction Treatment during 2014-2016 who Used Heroin or Prescription Opioids in the Past Year or who Misused Prescription Opioids in the 30 Days Prior to Jail, Prison, or Attending Treatment

	**Men (n=288)**		**Women (n=135)**	
**Exposure**	Bivariate PR (95% CI)	Adjusted[a] PR (95% CI)	Bivariate PR (95% CI)	Adjusted[a] PR (95% CI)
**Experienced an Overdose**	**2.3 (1.6-3.3)**	**1.5 (1.1-2.2)**	**2.0 (1.2-3.1)**	1.4 (0.82-2.3)
**Witnessed an Overdose**	**1.5 (1.1-2.1)**	1.2 (0.93-1.6)	1.5 (0.96-2.4)	1.3 (0.91-1.8)
**High Justice Involvement Class**	0.88 (0.71-1.1)	1.1 (0.87-1.3)	0.89 (0.72-1.1)	0.99 (0.80-1.2)

[a]Adjusted for age, race, education level, temporary housing (defined as reporting living in a halfway house or group home, inpatient facility, jail, shelter, or homeless), heroin use (in the past year), and injection drug use (in the 30 days prior to attending treatment, jail, or prison). Abbreviations: PR: Prevalence Ratio, 95% CI: 95% Confidence Interval.
